# Triple-negative breast cancer treatment with core-shell Magnetic@Platinium-Metal organic framework/epirubicin nano-platforms for chemo-photodynamic based combinational therapy: A review

**DOI:** 10.1097/MD.0000000000039845

**Published:** 2024-09-27

**Authors:** Kangjie Xu, Yanhua Zhang, Hui Cheng, Weipeng Chen, Cheng Chen, Minglei Zhang, He Song, Feng Wang

**Affiliations:** a Department of Central Laboratory, Binhai County People’s Hospital, Yancheng, China; b Department of Obstetrics and Gynecology, Binhai County People’s Hospital, Yancheng, China; c Department of General Surgery, Binhai County People’s Hospital, Yancheng, China; d Department of Oncology, Binhai County People’s Hospital, Yancheng, China; e Department of Rehabilitation Medicine, Kanda College of Nanjing Medical University, Lianyungang, China.

**Keywords:** breast cancer, chemotherapy, epirubicin, metal-organic frameworks, photodynamic

## Abstract

The combination of chemotherapy and photodynamic therapy (PDT), enabled by core-shell nano-platforms, is a promising method to improve cancer therapy by overcoming hypoxia and boosting drug penetration in breast tumor. Core-shell magnetic (iron oxide: Fe_3_O_4_)@platinum-metal organic framework/epirubicin (abbreviated as M@Pt-MOF/EPI) nano-platform is considered an effective cancer therapeutic agent. Relatively small particle size, round shape, and specific response to pH, are the key features of these nanomaterials to be used as promising therapeutic agents. Chemotherapy and photodynamic therapy, when applied in addition to the anticancer effects of nanomaterials, further enhance the therapeutic efficacy. The extensive use, utilization, and efficacy of Core-Shell Magnetic@Platinium-Metal Organic Framework/epirubicin Nano-Platforms for chemo-photodynamic combination therapy in the treatment of several cancers, including triple-negative breast cancer, are examined in this in-depth investigation.

## 1. Introduction

Breast cancer is one of the leading cancer types in women, which poses a high burden on health systems across the globe. Among the subtypes of breast cancer, triple-negative 4TI breast tumor has been reported as the most aggressive cancers, which have a high mortality and morbidity rates.^[1,2]^ Due to the high drug resistance, higher chances of recurrence, poor prognosis, and great risk of metastasis, current treatment options such as surgery and chemotherapy available for triple-negative breast cancer,^[1]^ are not very promising and effective. Therefore, new technologies are required to be applied for better treatment of triple-negative breast cancer. One of the most effective treatment options can be the use of nano-platform for drug delivery. These nanoplatforms can help in effective combinational therapeutic strategies.^[3,4]^ To select the most suitable option, it is necessary to confirm the nature and characteristics of nanomaterials therefore, in the case of triple-negative breast cancer, metal nano-platforms can be of great importance such as using iron oxide (Fe_3_O_4_) with various coatings, considering their importance such as high loading capacity, biocompatibility, imaging capability, controlled catalytic activities, multimodal cancer treatment, feasible production, and target specific delivery.^[5–10]^ However, magnetic nanoparticles face a number of challenges, including uneven size distribution, limited drug loading, the use of hazardous chemical compounds in manufacturing, slow degradation with high agglomeration, and accumulation in vital organs with serious consequences. According to a number of studies, adding metal-organic frameworks (MOF) coatings on metal nano-platforms increases their porosity and functionality while also allowing for regulated drug release in an acidic environment.^[11–16]^ Additionally, MOF coating improves the nano-platforms’ biocompatibility, catalytic activity, and colloidal stability.^[17–21]^ In addition to increasing the stability of the drug and enhancing its permeability in cell lines related to triple-negative breast cancer, Chowdhuri et al^[22]^ and Alijani et al^[23]^ used nanoplatforms by using different cell lines and found that using M@IRMOF-3/folic acid in NIH3T3 and HUVEC cells has greater biocompatibility and thus can be used as a potential treatment option. Moreover, M@MOF-doxorubicin (Dox)-carbon-aptamer and arene-based metal complexes were also used against triple-negative breast cancer.^[2]^ It is now widely being studied that hyperthermia if combined with chemotherapy may increase the permeability of drugs as well as enhance the controlled release of Dox (drugs). In this regard, Chen et al^[24]^ demonstrated that a nanoplatform known as M@poly[dopamine]@ZIF-90/Dox showed higher effectiveness and thereby inhibited the development and proliferation of cancer cells as compared to free drugs. Moreover, Xiang et al^[25]^ treated breast cancer mice model with M@C-poly[vinylpyrrolidone] @Dox and found that this nanomaterial was more effective as compared to free Dox and thus significantly reduced the tumor size mediated by controlled pH-sensitive drug release. It also reduced drug toxicity in other organs and tissues, and increased biocompatibility as compared to the use of free Dox for the same purpose. Photodynamic therapy in combination with chemotherapy can kill tumor cells through the utilization of single oxygen molecules (^1^O_2_) and thus can restrict the growth of cancer cells further. Hence, this combinational therapeutic option can be more effective against triple-negative breast cancer cells as well as effectively overcome hypoxia as compared to the therapeutic option with a single modality approach. For example, Pandey et al^[26]^ and Zhang et al^[27]^ via a combination of chemotherapy with PDT enabled by Fe-TBP and PFTBA@HSA-DVDM@PM nano-platforms, respectively, were able to inhibit the proliferation of colorectal and breast malignancies up to 70% and 90% mediated by boosting the generation of ^1^O_2_ in tumor tissues. However, their findings suggest that the O_2_ supply in malignant tissues is insufficient to generate a significant amount of ^1^O_2_. While using MOF-coated nano-platforms, it remains impractical to control the extent and mechanism of toxicity in healthy and cancerous tissues, the rate of anti-hypoxia effects via catalytic properties, the rate of drug accumulation in targeted sites, and the extent of drug penetration. Although combining PDT with M-based MOFs showing peroxidase (POD)-mimic activity can increase the generation of reactive oxygen species (ROS) for cancer therapeutics, it appears that using other nanomaterials as MOF modifiers such as platinum (Pt), gold, or silver can increase the combinatory cancer therapeutics through dual enzyme-mimic activity.^[28,29]^ Accordingly, it has been demonstrated that the use of Pt nanoparticles on Fe_3_O_4_ nano-platforms significantly improves breast cancer therapeutics via an increase in ROS generation enabled by POD-mimic activity.^[30]^ Also, it has been revealed that the M-Pt nano-platforms have CAT-mimic activity, which is very effective in overcoming hypoxia and increasing drug permeability in cancer tissue.^[30,31]^ Similarly, Li et al^[32]^ indicated that the application of Pt-modified Fe_3_O_4_ nano-platforms with photo-enhanced dual POD- and CAT-mimic activity selectively inhibited pancreatic cancer cell proliferation. The drug’s penetration into tumor tissue, as well as the accumulation of ROS and ^1^O_2_ in the tumor microenvironment, have the potential to significantly improve cancer therapeutics thanks to the integration of Fe_3_O_4_ and Pt nanoparticles.

The widely known anticancer drug “epirubicin” is a Dox epimer presented with a 4’-hydroxyl group inversion on sugar molecules.^[33]^ The primary mechanism of action associated with epirubicin is expressed by RNA or DNA damage induced by the activity of suppressed topoisomerase II. In addition, this DNA or RNA damage is caused by downregulation of protein synthesis, radical production, and disruption of the cell membrane, thereby leading to cell cycle arrest in order to prevent cell division or growth or induce apoptosis.^[34]^ Comparatively, epirubicin is considered less harmful than Dox, however, we cannot rule out the possibility of serious side effects including non-hematological and cardiac toxicities, and myelotoxic activities.^[35]^ It is also important to note that increased dosage and long-term use is required in order to boost the effectiveness or epirubicin, which in turn increases the risk of cardiac toxicity as well as pose the related side effects.^[36]^ However, the efficacy of epirubicin in treating stomach, neuroblastoma, liver, squamous cell, lung, and colorectal cancers cannot be neglected and therefore, its use in the development of anticancer platforms or nano-drugs is highly recommended. It has been widely studied and confirmed that nanomaterials or nano-platform can help reduce toxicity while increasing the efficacy of epirubicin in treating different types of cancers. In this regard, De Vita et al^[37]^ recently demonstrated the use of lipid nanovesicles in combination with epirubicin. This combination successfully lessened the growth of triple-negative breast tumor and increased the biocompatibility and efficiency of nanoparticles with reduced toxicity as compared the free epirubicin. However, there are still some challenges associated with the dose and duration of EPI use, drug loading and release levels, toxicity in off-target tissues, EPI permeability, cancer tissue hypoxia levels, and drug distribution throughout the body.^[38–42]^ The use of smart porous core-shell M@Pt-MOF/EPI nano-platforms provides a possible therapeutic approach for triple-negative breast cancer and additionally incorporation of photodynamic therapy is required to maximize its promise as alternative treatment.

## 2. Synthesis of nanomaterials

In order to fabricate Fe_3_O_4_ nano-platforms, ethylene glycol, and diethylene glycol are added with iron chloride, sodium acrylate, and sodium acetate. After mixing, the obtained homogeneous solution is heated in a Teflon-lined stainless-steel autoclave at 220°C and the Fe_3_O_4_@poly[acrylamide] nano-platforms are separated and washed with ethanol and water, followed by sample drying in a vacuum for 24 hours. The aqueous Fe_3_O_4_@poly[acrylamide] solution was diluted with water and ethanol, then sonicated and treated with ammonia solution. A solution of tetraethyl-orthosilicate in ethanol was injected, and the end product was magnetically obtained 2 hours later. Fe_3_O_4_@poly[acrylamide]@SiO_2_ samples were washed, vacuum dried for 1 day, heated at 650°C for 5 hours, mechanically shaken with NaOH for 7 hours, and then magnetically collected and washed with water and ethanol.

Platinum chloride and terephthalic acid were mixed with anhydrous ethanol and N-dimethylformamide to produce Pt-MOF. Fe_3_O_4_ nano-platforms dissolved in ethanol were added, followed by agitation and autoclaving at 150°C for half a day. The product was then cooled, cleaned with ethanol and water 3 times, and dried at 80°C. At pH 8.0 and room temperature, nanocarriers were loaded onto core-shell M@Pt-MOF nano-platforms through EPI incubation. Thermogravimetric analysis, for size distribution and zeta potential, N_2_ adsorption for surface gaps, X-ray diffraction for crystalline structure, and Fourier transform infrared spectroscopy for chemical composition assessment were all used in the characterization.

## 3. Fe and drug distribution

When core-shell M@Pt-MOF/EPI nano-platforms were injected into tumor tissue 12 hours after the controls, Fe permeability and retention were significantly higher, as demonstrated by an analysis of Fe accumulation and bio-distribution. The spleen’s Fe concentration increased as a result of core-shell M@Pt-MOF/EPI nano-platforms, but the liver, brain, or kidney’s Fe levels remained the same, indicating a possible decrease in clearance. Significant EPI targeting to tumor locations was also seen, with decreased concentrations and higher accumulation in off-target tissues, which was corroborated by histological investigation and supported a reduction in inaccurate side effects.

## 4. Efficiency of EPI on nanoparticles

It has been well established that epirubicin is an effective and efficient drug that can be used against tumor, however, the efficacy of this drug is low when given in low concentrations or low doses.^[36]^ Moreover, we know that long-term use of this drug (epirubicin) at high concentrations and high dosage is generally recommended to get significant effect and high effectiveness. However, using this drug at high concentrations causes high levels of toxicity in different organs and tissues such as bone marrow, brain, kidney, liver, and gastrointestinal tract.^[43]^ In recent years, researchers have developed and reported a number of promising nanocarriers that can be used to deliver epirubicin to target tumors.^[37,41,42,44]^ Using typical nanocarriers the loading efficiency of epirubicin is limited, therefore, an efficient nano platform is required for enhanced efficacy and efficiency. The use of core-shell from metal oxide nanoparticles in combination with chemical compounds can be a suitable nano-platform. These nano platforms contain dynamic properties including release control, high drug loading capacity, reduced agglomeration, reduced toxicity, target specific, easy to introduce, and the potential of high effectiveness in auxiliary cancer.^[45–47]^ Typically, the hydrothermal and layer-by-layer methods are used to create the core-shell M@Pt-MOF/EPI nano-platforms, for instance, Ebrahimi et al^[48]^ and Chen et al^[21]^ reported the efficacy of core-shell M@Pt-MOF/EPI nano-platforms on cancer. In addition, it is convenient to manufacture such as the layer-by-layer or multi-stage manufacturing process is reliable.^[49,50]^

## 5. Significance of core-shell M@Pt-MOF/EPI nano-platforms

According to Laha et al^[51]^ core-shell M@Pt-MOF/EPI nano-platforms have a significant efficacy in improving mice weight and reducing the weight of TNFB tumors when compared to free EPIRUBICIN and also with the in vivo results. Also consistent with previous findings,^[52,53]^ it was discovered that combining chemotherapy and PDT with increasing intracellular ROS generation via the developed platform’s POD-mimic activity has a significant effect on TNFB tumor weight loss. However, Xiang et al^[25]^ displayed that the core-shell nano-platforms do not make a significant difference in the weight of mice compared to the free drug. In addition, in line with the results of Xiang et al^[25]^ and Liu et al,^[54]^ the anti-tumor efficacy of core-shell M@Pt-MOF/EPI nano-platforms is attributed to increased Fe retention and high EPIRUBICIN accumulation in TNFB tissue. Therefore, according to the findings of Yen et al^[55]^ the use of core-shell M@Pt-MOF/EPI nano-platforms effectively overcomes tumor tissue hypoxia by producing O_2_ via possible H_2_O_2_ degradation in tumor tissue. In fact, it has previously been demonstrated that increasing drug permeability to solid tumors reduces tumor size and is attributed to the generation of O_2_.^[56,57]^ Cheng et al^[58]^ shows that the combination of chemotherapy and PDT enabled by core-shell M@Pt-MOF/EPI nano-platforms overcomes hypoxia toward the enhancement of tumor therapy more effectively than using bare nano-platforms. In this regard, Ma et al^[59]^ and Yang et al^[60]^ found that the loading of drugs onto hypoxia-overcoming nanoparticles such as Pt and tin can enhance single oxygenation in tissue and increase drug permeability. Furthermore, in agreement with Xiang et al^[25]^ histological analyses demonstrated that core-shell M@Pt-MOF/EPI nano-platforms cause no toxicity to main tissues.^[61]^ So, treating the TNFB tumors with core-shell M@Pt-MOF/EPI nano-platforms appears to be a promising approach. This is explained by their high drug loading capacity, accurate pH-sensitive drug release control, chemo-photodynamic combination therapy application, and capacity to increase drug toxicity in TNFB cells while minimizing toxicity to vital organs.

## 6. Conclusion

In general, M@Pt-MOF nano-platform with a core-shell structure containing EPIRUBICIN was synthesized by hydrothermal and layer-by-layer methods. The results of, TGA, DLS, N_2_ adsorption, XRD, and FTIR analyses confirmed a spherical shape of nano-platforms with a diameter of 45 to 85 nm and high porosity on which Pt-MOF and EPIRUBICIN are loaded. In addition, it was shown that the presence of Pt-MOF enhances the POD- and CAT-mimic activity of the core-shell M@Pt-MOF nano-platforms. Also, toxicity analysis revealed that core-shell M@Pt-MOF/EPI nano-platforms, despite their good biocompatibility against NIH3T3 cells, have high toxicity against TNFB cells by inducing apoptosis mediated by high levels of intracellular ROS. Furthermore, pH-responsive release of the EPIRUBICIN by core-shell M@Pt-MOF/EPI nano-platforms and reduction of EPI toxicity in off-target tissues are the potential advantage of the core-shell M@Pt-MOF/EPI nano-platforms, which ultimately resulted in a significant reduction of TNFB tumor size through overexpression of HIF-1α and Caspase-3 mRNA to overcome tumor hypoxia and induce apoptosis, respectively. Moreover, in vivo observations illuminate that the core-shell M@Pt-MOF/EPI nano-platforms have high potential in chemo-photodynamic combination therapy for improving TNFB tumor treatment. So the Nano-Platforms design that the use of core-shell M@Pt-MOF/EPI nano-platforms as promising nanoparticle-based drug delivery systems for cancer therapy.

## Acknowledgments

The current study was supported by the Research Development Fund of Kanda College of Nanjing Medical University (KD2023KYJJ163).

## Author contributions

**Conceptualization:** Kangjie Xu, He Song, Feng Wang.

**Data curation:** Yanhua Zhang, Weipeng Chen, He Song.

**Formal analysis:** Cheng Chen.

**Investigation:** Yanhua Zhang, Weipeng Chen.

**Methodology:** Hui Cheng.

**Project administration:** Yanhua Zhang.

**Resources:** Hui Cheng.

**Software:** Cheng Chen.

**Supervision:** Feng Wang.

**Validation:** Hui Cheng.

**Visualization:** Cheng Chen.

**Writing – original draft:** Kangjie Xu, Yanhua Zhang, Weipeng Chen, Minglei Zhang.

**Writing – review & editing:** Hui Cheng, Cheng Chen, Minglei Zhang, He Song, Feng Wang.
